# Digital competency mapping dataset of pre-service teachers in Indonesia

**DOI:** 10.1016/j.dib.2023.109310

**Published:** 2023-06-13

**Authors:** Muhammad Luthfi Hidayat, Hariyatmi, Dwi Setyo Astuti, Bambang Sumintono, Maram Meccawy, Tariq J.S. Khanzada

**Affiliations:** a Information System Department, King Abdulaziz University, Saudi Arabia; b Biology Education Department, Universitas Muhammadiyah Surakarta, Indonesia; c Faculty of Education, Universitas Islam Internasional Indonesia, Indonesia

**Keywords:** Digital competency, Pre-service teachers, Rasch model, Digital literacy, Teacher training

## Abstract

This dataset used the Digital Competency Scale (DCS) to describe Indonesian pre-service teachers’ perceptions. The DCS instrument consisted of five constructs/dimensions, which are: 1) data and information literacy, 2) communication and collaboration, 3) digital content creation, 4) safety, and 5) problem-solving, with a total of 36 items using five-point agreement Likert scale. The data was gathered from 23 education and teacher training faculties at Muhammadiyah Universities in 14 provinces across Indonesia in the academic year 2021/2022. A total of 1400 students (18 to 23 years old) in their first to fifth years of study were recruited using the convenience sampling technique, where they participated in filling in the survey electronically using Google Form. The dataset was analysed with the Rasch model measurement approach using WINSTEPS version 5.2.3 software for data cleaning and validation, and reliability and validity testing of the instrument. This dataset analysis can help teacher-training institutions, or higher education policymakers design effective programmes to improve pre-service teachers' digital competencies. Furthermore, researchers can compare this dataset with more rigorous data from other countries.

**Specifications Table**

**Table:** 

Subject	Social science
Specific subject area	Digital competency, technology in education, teacher's competency
Type of data	Table Graph Figure
How data was acquired	The data was acquired through an online survey. The data was imported to Microsoft Excel, and analysed using Rasch model measurement software.
Data format	Raw Analysed Descriptive
Description of data collection	Primary data was gathered by a Likert-type scale questionnaire (Google Form) from 1400 students in their first to fifth year. The respondents were recruited using convenience sampling technique in the academic year 2021/2022.
Data source location	Data was collected from 23 faculties of education and teacher training from a private university network in 14 provinces across Indonesia.
Data accessibility	Dataset was uploaded to Mendeley Data Repository name: Mendeley Data identification number:10.17632/5kwtxjrbzg.3 Direct URL to data: https://data.mendeley.com/datasets/5kwtxjrbzg

## Value of the Data

• This dataset is valuable for policymakers in establishing teacher education programmes that assist pre-service teachers in comprehending their motives and attempts to enhance their knowledge, abilities, and experiences in digital competency.
• Due to the Covid-19 pandemic that had shifted learning processes to encourage digital technology integration in Indonesia, the data gathered would meet the demand for digital competence among teachers, and prospective teachers.
• The data is helpful for researchers who want to compare the results of this study to similar research related to digital literacy or digital citizenship frameworks in seeking and finding solutions to overcome obstacles related to digital technology integration in the higher education context using other statistical analyses.
• This dataset on pre-service teachers in Indonesia can provide valuable information in mapping and predicting the progress of information technology-based education in the country with the largest population in Southeast Asia.
• Due to teaching concerns, this dataset can be used by lecturers to train their students about data pre-processing using Rasch model measurement.

## 1 Objective

After the Covid-19 pandemic had disrupted the way education works, university systems have used the Information and Computer Technology (ICT) in many aspects of teaching and learning. Therefore, many countries have recently been developing national and international policies to improve and support digital competencies [1]. In teacher training for higher education, the need for digitally literate teachers has increased significantly because the graduates must have the ability to enhance their students’ digital competencies, and use digital technologies in the learning-teaching process effectively in the future [2]. This dataset is influential for assessing digital competency in several aspects, such as data and information literacy, communication and collaboration, digital content creation, safety, and problem-solving. According to the literature, research on mapping these variables with respondents from various backgrounds at the national level needs to be made more apparent. The higher education policymakers also need brief information to design courses or training programmes that meet the demand for digitally competent teachers. Previous studies emphasised the implementation and importance of this competency in several developing countries [3,4]. However, only a few published studies investigate how to map pre-service teachers’ digital skill potential in a vast country. Furthermore, the data can inform the formulation of action plans, decision-making considerations, or interventions that best support teacher training and education programmes.

## 2 Data Description

For that reason, this paper presents a dataset that describes the competency map of Indonesian pre-service teachers from the first to fifth study years (between ages 18 to 23) using the Digital Competency Scale (DCS), which follows the DigComp framework [5], with several demographic information added. The data was divided into two groups: (1) demographic information, including gender, region, year of study, and department; and (2) the determinant competencies, which consist of the dimensions of data and information literacy, communication and collaboration, digital content creation, safety, and problem-solving. A total of 36 items of a 5-point Likert-type scale were used to measure the knowledgeability of respondents as a form of digital competency mapping. Originally, there were 1400 pre-service teachers from 14 provinces in 6 big islands in Indonesia who participated in the study electronically using Google Form.

The Rasch model is an analysis model used in this study. It has been widely employed across diverse domains, including education, commerce, psychology, healthcare, and other disciplines within the social sciences. The model is suitable for measuring latent traits in assessing human opinions, perceptions, and attitudes [6]. The Rasch analysis provides several statistical analytics: descriptive analysis, Chi-square (χ2), unidimensionality of rating scale, person and item reliability, and Cronbach Alpha index. The current analytical model provides a thorough methodology for clarifying the degrees of item complexity via precise and meticulous measurement, commonly referred to as item calibration [7]. The utilization of a conjoint-measurement technique is implemented in order to calibrate a measurement model that establishes the correlation between an individual's ability and the difficulty level of an item. This is achieved through the use of a logit (logarithm odd unit) scale as a standardized unit of measurement, as outlined by Linacre [8]. The first stage of analysis was conducting data cleaning and validation using WINSTEPS, a Rasch measurement model software, to detect outliers (responses with extreme maximum and minimum values), and misfits (responses for having an Outfit MNSQ index larger than 2.0) [5]. In the end, 1264 responses were further analysed, showing adequate data stability far beyond the minimum requirement for any sampling size method. The tables below depict respondent analytics before (Table 1 with 1400 respondents) and after data cleaning (Table 2 with 1264 respondents).

**Table 1:** Summary of Statistics of N=1400 Respondents (Pre-Data Cleaning)

					Infit		Output	
	Total score	Count	Measure	Model error	MNSQ	ZSTD	MNSQ	ZSTD
Mean	138.8	36.0	1.61	.29				
Standard Deviation	19.2	.0	1.44	.25				
Max.	180.0	36.0	7.37	1.83				
Min.	53.0	36.0	-2.75	.21	.16	-6.4	.16	-6.2

Real RMSE	.40	True SD	1.38	Separation	3.43	Reliability	.92	
Model RMSE	.38	True SD	1.39	Separation	3.65	Reliability	.93	

Standard Error of person Mean = .04

Cronbach Alpha person raw test reliability = .95

**Table 2:** Summary of Statistics of N=1264 Respondents (Post-Data Cleaning)

					Infit		Output	
	Total score	Count	Measure	Model error	MNSQ	ZSTD	MNSQ	ZSTD
Mean	138.8	36.0	1.95	.27	1.01	-.3	1.00	-.3
Standard Deviation	19.2	.0	1.29	.06	.50	2.4	.49	2.3
Max.	180.0	36.0	6.92	1.01	2.75	5.3	2.71	5.2
Min.	53.0	36.0	-3.59	.25	.16	-6.4	.16	-5.9

Real RMSE	.32	True SD	1.25	Separation	4.13	Reliability	.94	
Model RMSE	.28	True SD	1.26	Separation	4.56	Reliability	.95	

Standard Error of person Mean = .04

Cronbach Alpha person raw test reliability = .95

As shown, the person logit mean went from 1.61 logit (standard deviation, SD 1.44) (table above) to 1.95 logit (SD 1.29) after data cleaning (table below), which was an increase of 0.3 logit scale. This shows that all respondents perceived themselves as having a higher level of digital competency with a standard deviation higher than 1.0, indicating a very wide dispersion level among the respondents. However, the data quality in Table 2 showed better reliability, where the person separation index became 4.13, and person reliability rose to 0.94. Furthermore, Table 2 indicates that the data fits to the model, where the mean values of Infit and Outfit MNSQ are close to ideal 1.0, but are not able to be revealed in Table 1 since there are outliers.

Table 3 presents the characteristics of the respondents based on the demographic information after the data cleaning. The sample of this study was dominated by female students (82.5%). The biggest portion of participants were in their second and third year of study (736 students, 58.2%), with a large majority of them majoring in social studies (42.2%), followed by language (29.8%), mathematics and science (23.2%), and vocational (4.8%). Based on the students’ origin, 800 were from the region of Java (63.3%), then Sulawesi and Maluku (11%), Nusa Tenggara (10.8%), Sumatera (11.6%), Kalimantan (2.4%), and Papua (0.9%).

**Table 3:** Demographic Profile of Respondents (N=1264)

Demographic Variable	Category	Frequency	Percentage (%)
Gender	Male	229	17.5
	Female	1035	82.5
	N	1264	100

Year of Study	1^st^	238	18.8
	2^nd^	335	26.5
	3^rd^	401	31.7
	4^th^	281	22.2
	5^th^	9	0.8
	N	1264	100

Department of Education	Math & Science	293	23.2
	Vocational	61	4.8
	Social Studies	532	42.2
	Language	377	29.8
	N	1264	100

Region	Sumatera	147	11.6
	Java	800	63.3
	Kalimantan	30	2.4
	Sulawesi & Maluku	139	11
	Nusa Tenggara	137	10.8
	Papua	11	0.9
	N	1264	100

Table 4 shows the two-facet item and person rating scale model processed for the 36 Digital Competency Scale (DCS) items, and 1264 respondents using the Rasch rating scale model (RSM) approach. As shown in the table below, the mean measure (logit) of the items is 0.00 logit, and the standard deviation is considered good (0.56), suggesting that the dispersion of measures was wide across the logit scale in terms of the item difficulty level. For person, the logit mean was 1.95 logit, showing that all respondents tended to perceive themselves as having a higher level of digital competency, with a standard deviation of 1.29, indicating a very wide dispersion level among the respondents. The standard error value was small, informing that the measurement is precise and accurate in terms of measuring this digital competency variable. The raw variance is higher than 40%, and the chi-square statistics result was significant, showing a uniform fit to the model. The separation index (more than three) [6], and reliability (more than 0.9) [7] of the item and person statistics suggest very good reliability.

**Table 4:** Summary of Person and Item Statistics

	Person	Item
N	1264	36
Measure (logit)		
*Mean*	1.95	0.00
*Standard deviation, SD*	1.29	0.56
*Standard Error, SE*	0.03	0.10
Separation	4.13	14.13
Reliability	0.94	1.00
Cronbach's Alpha	0.95	
Raw variance	44.6%	
Chi-Square (X^2^)	85168.10*	

*p < 0.01

Table 5 presents the participants’ response to each construct of DCS, which indicates their knowledgeability of DigComp. The construct of *digital content creation* had the highest logit (0.505 logit), showing that students perceived that they are good at it, followed by the construct *problem solving*, and *communication and collaboration*, which can be considered as competent, but not really good. The last two constructs, which were *safety* (-0.321 logit), and *data and information literacy* (-0.352 logit), had negative logit mean values, indicating that the respondents can be mostly considered not really good in these two competencies.

**Table 5:** Respondents’ Knowledgeability of DigComp Based on Competency Area (N=1264)

Competency Area	Logit Mean	Standard deviation
Data and information literacy	-0.352	0.537
Communication and collaboration	0.041	0.795
Digital content creation	0.505	0.409
Safety	-0.321	0.607
Problem-solving	0.147	0.410

**Fig. 1:**
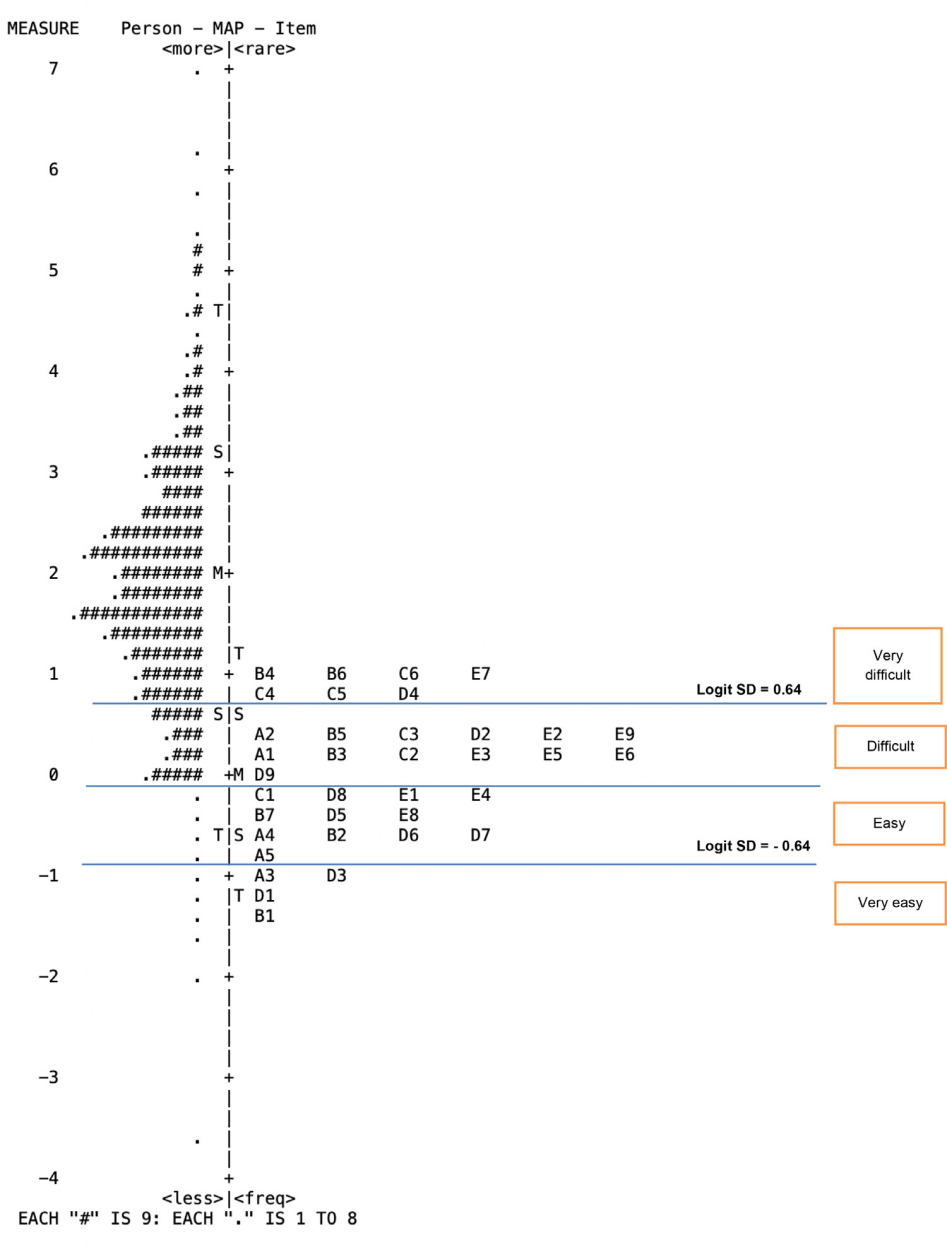
Item Wright Map to Show Competency Mapping According to Item Difficulty Level

Table 6 gives information about item difficulty by the mean value of each area, which presents the difficulty level of a specific competency area. The items in the DCS instrument were classified into four difficulty levels by dividing the distribution of the item logit scores based on mean (0.00), and standard deviation values (0.56), as shown in Table 4. A higher logit value item (LVI) indicates that the item has high-level difficulty with the respondents. In total, there were 7 items (19%) in the category of *very difficult*, as agreed to by respondents (LVI > 0.56 logit). In second was the category *difficult* (+0.56 > LVI > 0.00), for which there were 13 items (36%), the biggest number of items. In the next category, which is *easy* (0.00 > LVI > -0.56), there were 12 items (33%). Lastly, four items (11%) fell under the category of *very easy* by the respondents (LVI < -0.83 logit). Graphically, this is also shown in Fig. 1 below.

**Table 6:** Item Difficulty Level Classification According to Logit Value of Item (LVI)

			Logit Value Item			
Area	Code	Item	Very difficult LVI > 0.56 logit	Difficult +0.56 ≥ LVI ≥ 0.00	Easy 0.00 ≥ LVI ≥ −0.56	Very easy LVI < −0.56 logit
Data and information literacy	A1	I can use a search engine to limit the number of searches using a filter.		√		
	A2	I can identify search results based on novelty, validity, type, file format, or modify my search.		√		
	A3	I am used to comparing different sources of information to decide whether it is true.				√
	A4	I store and organise digital resources for personal use later.			√	
	A5	I understand the copyright rules that apply to digital resources that I use.			√	

Communication & collaboration	B1	I am used to using digital technology to explore, interact, or discuss to get updates on the world I work.				√
	B2	I can choose the right digital media to share and exchange digital content.			√	
	B3	I develop strategies for improving educational practice with digital technology, either individually, or collaboratively.		√		
	B4	I like to express my thoughts and opinions through relevant social media.	√			
	B5	I actively take advantage of the digital community I follow to collaborate on assignments.		√		
	B6	While working online, I understand netiquette, its application, and its impact on my reputation, career, and others.	√			
	B7	I understand the risks and threats to my identity in the digital environment, and how to mitigate them.			√	

Digital content creation	C1	I can develop digital content properly.			√	
	C2	I am proficient in using applications to develop relevant multimedia.		√		
	C3	I can modify and combine existing digital learning resources according to the competencies to be achieved.		√		
	C4	I understand the meaning and consequences of types of intellectual property.	√			
	C5	I am used to asking permission from the copyright owner before copying, or distributing content.	√			
	C6	I make use of, and create digital content, or at least be able to practise programming in solving problems.	√			
Safety	D1	I understand how to activate, utilise, and update security features on my device.				√
	D2	I understand the risks of cyber-attacks on the device/gadget I use.		√		
	D3	I am quite careful, and have good judgement about when to share (or not share) personal information, and sensitive data.				√
	D4	I know various methods for identifying phishing, and malware.	√			
	D5	I can encrypt, password-protect, or otherwise secure access to data as it is transmitted or stored.			√	
	D6	I notice technology-related physical symptoms (headaches, blurred vision, or wrist pain that may be signs of overuse).			√	
	D7	I am very concerned about maintaining a balanced use of technology.			√	
	D8	I understand cyberbullying, and how to deal with or fight it.			√	
	D9	I understand and practise using gadgets/devices healthily.		√		

Problem-solving	E1	I know how, step by step, to find a problem, and find a solution, and not be afraid to try some new tutorials.			√	
	E2	I am patient, tenacious, and not overly frustrated when technical problems arise.		√		
	E3	I can efficiently use advanced hotkeys for relevant applications.		√		
	E4	I can easily edit advanced settings on digital devices, online services, and applications.			√	
	E5	I understand well when technology can support a process (or cannot).		√		
	E6	I am passionate about creating or editing digital content.		√		
	E7	I can detect and fight plagiarism using digital technology.	√			
	E8	I try to improve and update my digital pedagogical competencies because of my limitations.			√	
	E9	I can use digital technology to provide advice or tutorials to colleagues on learning innovation practices.		√		
N			7	13	12	4

**Fig. 2:**
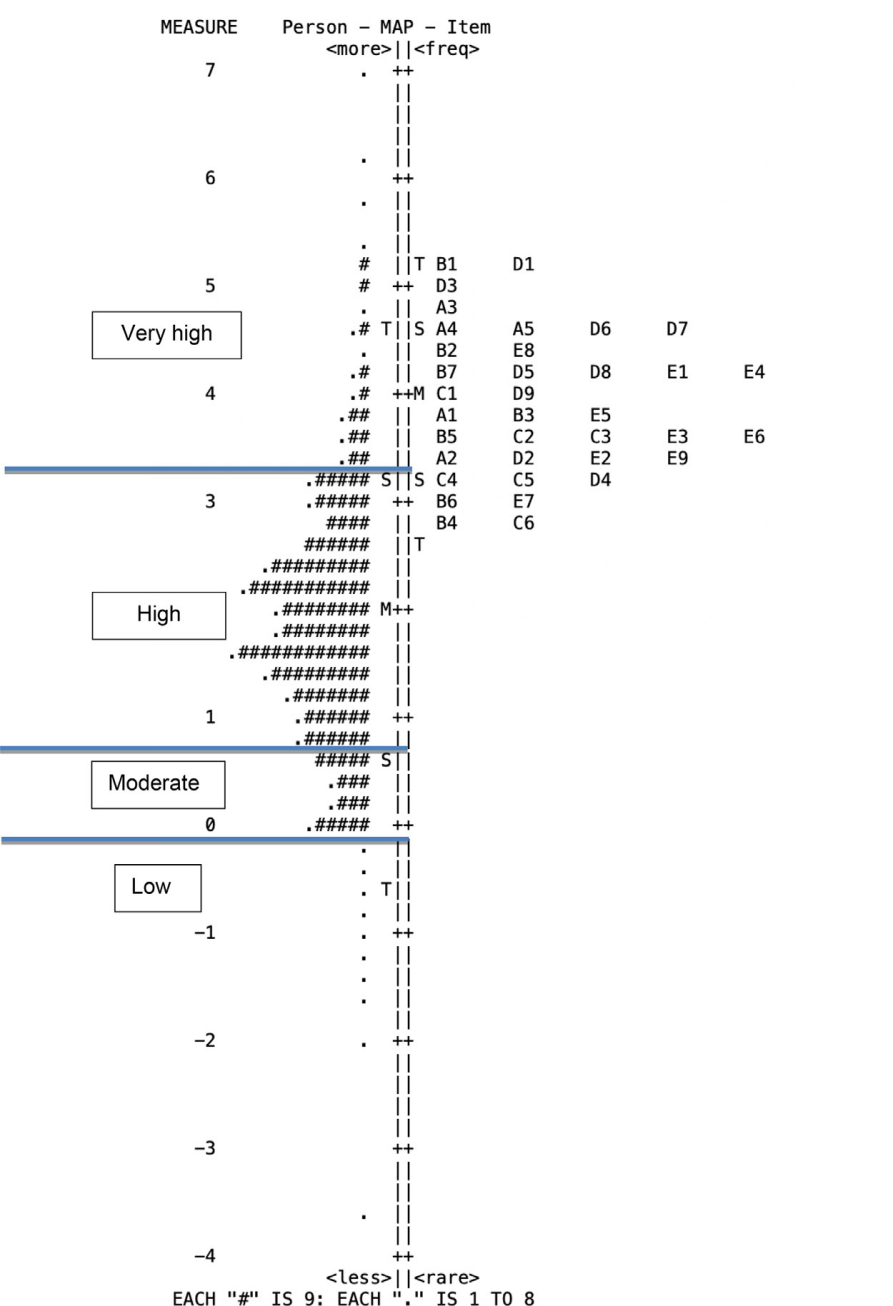
Item Person Map of Pre-Service Teachers’ DigCom Knowledge ability

Table 7 focuses on grouping participants’ responses based on their logit score. The person logits were classified into four levels of knowledgeability by dividing the distribution of the person logit mean score (1.95), and its standard deviation value (1.29), as appeared in Table 4. The distribution of the pre-service teachers’ knowledgeability with regard to digital competency based on demographic background is described by the logit value of the person (LVP) in Table 7 above, and Fig. 2 below.

**Table 7:** Logit Value of Persons (LVP) of Digital Competency (N=1264)

Demographic Variable	Very high level knowledgeability	High level knowledgeability	Moderate level knowledgeability	Low level knowledgeability
	LVP ≥ 3.24	1.29 < LVP < 3.24	0.88 < LVP <1.29	0.88 ≤ LVP
**Gender**				
Male	34 (19.2%)	84 (47.5%)	22 (12.4%)	37 (20.9%)
Female	143 (13.8%)	590 (57.1)	108 (10.5%)	192 (18.6%)
**Year of Study**				
1	32 (13.2%)	132 (54.6%)	32 (13.2%)	46 (19.0%)
2	44 (12.9%)	192 (56.6%)	33 (9.7%)	70 (20.7%)
3	53 (12.9%)	214 (52.3%)	54 (13.2%)	88 (21.5%)
4	46 (16.4%)	167 (59.6%)	27 (9.64%)	40 (14.3%)
5	2 (22.2%)	4 (44.4%)	1 (11.1%)	2 (22.2%)
**Region**				
Java	122 (16.3%)	443 (59.3%)	48 (6.4%)	134 (17.9%)
Sumatera & Babel	16 (11.0%)	77 (53.1%)	14 (9.7%)	38 (26.2%)
Kalimantan	3 (11.1%)	20 (74.1%)	1 (3.7%)	3 (11.1%)
Sulawesi & Maluku	20 (15.5%)	68 (52.7%)	15 (11.6%)	26 (20.2%)
Nusa Tenggara	16 (12.7%)	68 (53.9%)	17 (13.5%)	25 (19.8%)
Papua	-	5 (55.6%)	1 (11.1%)	3 (33.3%)
**Field of study**				
Math & science education	36 (12.7%)	160 (56.3%)	40 (14.1%)	48 (16.9%)
Social studies education	76 (14.9%)	289 (56.8%)	63 (12.4%)	81 (15.9%)
Language education	57 (13.9%)	227 (55.4%)	35 (8.5%)	91 (22.2%)
Vocational education	9 (2.3%)	363 (92.8%)	10 (2.6%)	9 (2.3%)

## 3 Experimental Design, Materials and Methods

This segment provides a description of the cross-sectional quantitative survey method employed for the study. The sample was 1400 undergraduate pre-service teachers from various fields of study from teacher training faculties in 23 private universities/higher education institutions (HEIs) on six big islands in Indonesia (Sumatera, Java, Kalimantan, Nusa Tenggara, Sulawesi, and Papua) during the academic year 2021-2022. Regarding ethical considerations, the students’ consent to participate in this study was sought before filling in the questionnaire.

Data was collected through an online questionnaire using the Digital Competency Scale *(DCS)* developed from the Digital Competency framework, or the DigComp framework for citizens, and the digital literacy framework put forth by the Indonesian Minister of Information and Communications [9]. The DCS contained four basic demographic questions (i.e., gender, year of study, field of study, and region), and 36 items in five constructs/dimensions that addressed various aspects of pre-service teachers’ digital competencies.

All collected data was inputted into a Microsoft Excel file, and checked by WINSTEPS version 5.2.3, a Rasch measurement model software for data validation and cleaning. 33 respondents had outlier responses (maximum or minimum ratings). Next, data cleaning was performed to identify inconsistency in the respondents’ answers, and there were 103 aberrant responses. These responses and outliers were removed from the data, so the final number of respondents was 1264. The Rasch measurement model approach was the WINSTEPS software, which mathematically transformed raw ordinal (Likert-type) data by calibrating item difficulties and person abilities. The transformation was based on the frequency of response, which appeared as probability to become logit (log odd unit) via the logarithm function, which assesses the overall fit of the instrument, and person fit [10]. Later, a measurement model was calibrated by conjoint measurement to determine the relationship between the item difficulty level, and the person's ability using the same unit scale, a scaled logit (logarithm odd unit) [8].

## Ethics Statement

The authors ensured that the respondents’ participation was strictly voluntary and anonymous. Regarding the data collection, the Research Ethics Committee of Universitas Muhammadiyah Surakarta was responsible for this project, and granted the approval code of *1238/HIT/FKIP/2021*.

All respondents were invited by email and text message to participate in the study. The first page of the online questionnaire stated that their participation would be strictly anonymous and voluntary to address any ethical concerns. Thus, by completing the questionnaire, the respondents had given their consent. Respondents could leave the study at any time, and for any reason, with no penalty and loss of benefits to which they were entitled, if any. The online survey was written anonymously to guarantee the confidentiality of their personal data.

## CRediT authorship contribution statement

**Muhammad Luthfi Hidayat:** Conceptualization, Writing – original draft, Formal analysis. **:** Conceptualization, Resources. **Dwi Setyo Astuti:** Investigation, Funding acquisition. **Bambang Sumintono:** Data curation, Methodology. **Maram Meccawy:** Writing – review & editing, Validation. **Tariq J.S. Khanzada:** Supervision, Validation.

## Declaration of Competing Interests

The authors declare that they have no known competing financial interests or personal relationships that could have influenced the work reported in this paper.

## Data Availability

Digital Competencies Mapping Dataset of Pre-service Teachers in Indonesia (Original data) (Mendeley Data).

## Appendix Supplementary materials

Image, application 1
