# Supplementary material for: Digital competency mapping dataset of pre-service teachers in Indonesia
Source: Data Brief. 2023 Jun 13;49:109310. doi: 10.1016/j.dib.2023.109310 (PMC10439268; doi:10.1016/j.dib.2023.109310)
Supplement: Supplementary file 1 [file mmc1.docx]

**Informed Consent Letter**

# Identification of Investigators & Purpose of Study

Dear respondent,

At this moment, you are being asked to participate in a research study by Muhammad Luthfi Hidayat, a researcher from Universitas Muhammadiyah Surakarta. This study aims to map the digital competency of undergraduate pre-service teachers in Indonesia. This study will contribute to the researcher’s completion of his PhD thesis at the Faculty of Computing and Information Technology, King Abdulaziz University, Saudi Arabia.

# Research Procedures

This study consists of a survey through a questionnaire that will be administered to individual participants in private universities in Indonesia. You will be asked to answer a series of questions related to digital competency knowledgeability.

# Time Required

Participation in this study will require approximately 15 minutes of your time.

# Risks

#### The investigator only perceives minimal risks from your involvement in this study (that is, no risks beyond the risks associated with everyday life).

# Confidentiality

The results of this research will be presented in an International scientific journal. While individual responses are obtained and recorded anonymously and kept in the strictest confidence, aggregate data will represent averages or generalisations about the responses. No identifiable information will be collected from the participant, and no identifiable responses will be presented in the final form of this study. All data will be stored in a secure location accessible only to the researcher. The researcher retains the right to use and publish non-identifiable data.

# Participation & Withdrawal

Your participation is entirely voluntary. You are free to choose not to participate. Should you choose to participate, you can withdraw at any time without consequences of any kind. However, once your responses have been submitted and anonymously recorded, you will not be able to withdraw from the study.

# Questions about the Study

If you have questions or concerns during the time of your participation in this study or after its completion, please contact:

Muhammad Luthfi Hidayat Tariq Jamil Saifullah Khanzada

Faculty of Teacher Training& Education Department of Information Systems

Universitas Muhammadiyah Surakarta King Abdulaziz University

[m.luthfi@ums.ac.id](mailto:m.luthfi@ums.ac.id) [tkhanzada@kau.edu.sa](mailto:tkhanzada@kau.edu.sa)

# Giving of Consent

I have read this cover letter and understand what is being requested of me as a participant in this study. I freely consent to participate. I have been given satisfactory answers to my questions. I certify that I am at least or more than 18 years of age.

___________________________

______________________________________

(Signed) 2021

The Research Ethics Committee has approved this study,

Reg. number # 118/A.3-III/FKIP/IV/2021

**The Digital competency Framework-based Questionnaire (DFBQ)**

Gender : (Male/ Female)

Year of Study : (1/2/3/4/5)

Department :

University :

Living Area : (Village/ Sub Urban/ City)

District/ Province :

| Area | Code | Item | Scale | | | | |
| --- | --- | --- | --- | --- | --- | --- | --- |
|  |  |  | Strongly  Disagree  (1) | Disagree  (2) | Neutral  (3) | Agree  (4) | Strongly Agree  (5) |
| Data and information literacy | A1 | I can use a search engine to limit the number of searches using filter. |  |  |  |  |  |
|  | A2 | I can identify search results based on novelty, validity, type, file format or allow to modify. |  |  |  |  |  |
|  | A3 | I am used to comparing different sources of information to decide whether it is true. |  |  |  |  |  |
|  | A4 | I store and organize digital resources for personal use later |  |  |  |  |  |
|  | A5 | I understand the copyright rules that apply to digital resources that I use |  |  |  |  |  |
| Communication &Collaboration | B1 | I am used to using digital technology to explore, interact, or discuss to get updates on the world I work. |  |  |  |  |  |
|  | B2 | I can choose the right digital to share and exchange digital content |  |  |  |  |  |
|  | B3 | I develop strategies for improving Education practice with digital technology, either individually or collaboratively. |  |  |  |  |  |
|  | B4 | I like to express my thoughts and opinions through relevant social media. |  |  |  |  |  |
|  | B5 | I actively take advantage of the digital community I follow to collaborate on assignments. |  |  |  |  |  |
|  | B6 | While working online, I understand netiquette, its application, and its impact on my reputation, career, and to others. |  |  |  |  |  |
|  | B7 | I understand the risks and threats to my identity in the digital environment and how to prevent them. |  |  |  |  |  |
| Digital content creation | C1 | I can develop digital content properly |  |  |  |  |  |
|  | C2 | I am proficient in using applications to develop relevant multimedia |  |  |  |  |  |
|  | C3 | I can modify and combine existing digital learning resources according to the competencies to be achieved. |  |  |  |  |  |
|  | C4 | I understand the meaning and consequences of types of intellectual property. |  |  |  |  |  |
|  | C5 | I am used to asking permission from the copyright owner before copying or distributing it. |  |  |  |  |  |
|  | C6 | I make use of, create digital content or at least be able to practice programming in solving problems. |  |  |  |  |  |
| Safety | D1 | I understand how to activate, utilize, and update security features on my device. |  |  |  |  |  |
|  | D2 | I understand the risks of cyber-attacks on the device/gadget I use. |  |  |  |  |  |
|  | D3 | I am quite careful and have good judgment about when to share (or not share) personal information and sensitive data. |  |  |  |  |  |
|  | D4 | I know various methods for identifying phishing and malware. |  |  |  |  |  |
|  | D5 | I can encrypt, password protect, or otherwise secure access to data as it is  transmitted or stored. |  |  |  |  |  |
|  | D6 | I notice technology-related physical symptoms (headaches, blurred vision, or wrist pain that may be signs of overuse). |  |  |  |  |  |
|  | D7 | I am very concerned about maintaining a balanced use of technology. |  |  |  |  |  |
|  | D8 | I understand cyberbullying and how to deal with or fight it. |  |  |  |  |  |
|  | D9 | I understand and practice using gadgets/devices healthily. |  |  |  |  |  |
| Problem-solving | E1 | I know how, step by step, to find a problem and find a solution, and not afraid to try some new tutorials. |  |  |  |  |  |
|  | E2 | I am patient, tenacious, and not overly frustrated when technical problems arise. |  |  |  |  |  |
|  | E3 | I can efficiently use advanced hotkeys for relevant applications. |  |  |  |  |  |
|  | E4 | I can easily edit advanced settings on digital devices, online services and  applications. |  |  |  |  |  |
|  | E5 | I understand well when technology can support a process (or cannot). |  |  |  |  |  |
|  | E6 | I am passionate about creating or editing digital content. |  |  |  |  |  |
|  | E7 | I can detect and fight plagiarism using digital technology |  |  |  |  |  |
|  | E8 | I try to improve and update my digital pedagogical competencies because of my limitation. |  |  |  |  |  |
|  | E9 | 9). I can use digital technology to provide advice or tutorials to colleagues regarding learning innovation practices. |  |  |  |  |  |
| Hereby, I declare that information I give is true. (Yes) | | |  |  |  |  |  |
